# The challenges of front-of-package labeling in Brazil

**DOI:** 10.3389/fnut.2022.921421

**Published:** 2022-10-13

**Authors:** Janine Giuberti Coutinho, Ana Carolina Feldenheimer da Silva, Inês Rugani Ribeiro de Castro, Elisabetta Gioconda Iole Giovanna Recine, Glenn Makuta, Nayara Cortês Rocha, Paula Johns, Raphael Barreto da Conceição Barbosa

**Affiliations:** ^1^Alliance for Adequate and Healthy Diets, Brasília, Brazil; ^2^Brazilian Institute of Consumer Protection (Idec), São Paulo, Brazil; ^3^Department of Social Nutrition, Rio de Janeiro State University (UERJ), Rio de Janeiro, Paraná, Brazil; ^4^Observatory on Food Security Policies and Nutrition (OPSAN), Brasília University (UnB), Brasília, Brazil; ^5^Slow Food Brazil, São Paulo, Brazil; ^6^Food the Right to Food and Nutrition Brazil, Brasília, Brazil; ^7^ACT Health Promotion, Rio de Janeiro, Brazil; ^8^Desiderata Institute, Rio de Janeiro, Brazil

**Keywords:** politics, food regulation, food label, food rights, food and nutrition security

The Brazilian legal system has plenty of mechanisms to ensure the public right to access clear information. The Federal Constitution (FC), enacted in 1988 and known as the “Citizen Constitution,” has as its principle to guarantee the right to human dignity. Protection of consumers, as well as their inviolable right to life and safety, are guaranteed by the FC as basic rights. The FC limits the country's economic order when establishing that its goal is to provide dignity for all Brazilians. Therefore, economic development cannot put people at risk, and its processes must be aligned with principles of goodwill and balance between consumers and suppliers. Collectively, FC establishes that health, food, and youth protection are social rights; hence, the government is responsible for providing policies for its implementation (1).

Due to the increase of obesity and non-communicable diseases (NCD), the actions related to food regulation are crucial to tackle the raising of malnutrition in all its forms, including the consequences of overweight, undernutrition, and micronutrient deficiency. Food regulation could be understood as a set of actions, led by the state, to protect consumers. As regulation actions could be listed the food taxation, the food marketing restriction/prohibition and the food labeling, including front-of-package nutrition labeling (FoPNL).

Improvements of food labeling permeate the discussion of food and nutrition security (FNS), considering the need to support the population on their food choices, even helping them understand the confusing information in the packages. That is also a part of consumers' rights. A label of easy comprehension contributes to the enforcement of the basic right of clear information on the composition and the risks of food products, as stated by the Brazilian Consumer Defense Code (CPC) (2).

At an international level, there are countless recommendations for governments to promote practices that regulate food labeling and inform consumers about the risks of the consumption of some products to their health, and facilitate their food choices, as stated in many international documents by the World Health Organization (WHO) (3), Food and Agriculture Organization of the United Nations (FAO) (4), and Pan American Health Organization (PAHO) (5).

The urgency and priority given to these subjects due to the current situation of health and nutrition of the Brazilians, a scenario that combines high levels of obesity and NCD (6), could be associated with factors related to the national rules of food labeling that may allow association with deceptive marketing and non-clear information, impacting on the growth of unhealthy foods consumption. Besides that, reading and comprehending nutrition facts panels are intricate for consumers, especially the interpretation of numbers and technical information, the need for calculations, small font size, and package visual pollution (7).

Evidence from Brazil aligned with international experiences, and society mobilization triggered political discussions. In 2013, the National Council for Food and Nutrition Security (*Conselho Nacional de Segurança Alimentar e Nutricional*- Consea) manifested for the first time that the National Health Surveillance Agency (*Agência Nacional de Vigilância Sanitária*- Anvisa) should provide “efficiency of the update and qualification of regulatory ideas for food labels,” as the entity is responsible for the food labeling standards in the country (8).

In 2014, Anvisa created a working group (WG) to discuss and elaborate regulatory proposals for the nutrition labeling of packaged food. The model of the FoPNL stood out as the main point of divergence among the proposals brought to light by Anvisa. Although the food industry, represented by the Brazilian Food Industry Association (*Associação Brasileira da Indústria de Alimentos*- ABIA), recommended the traffic-light labels, other proposals, aligned with the interests of public health, presented the model of warning labels with different formats. The civil society coalition “Alliance for Adequate and Healthy Diets” (“*Aliança pela Alimentação Adequada e Saudável*”) favored the model with the triangle format (9).

The warning labels have as their premise that the products with high content of nutrients related with obesity and NCDs must be easily identified to facilitate the healthiest food choice. Recent meta-analyses has shown that warning labels work better than colorful models (such as traffic-light and Nutri-Score) to discourage the purchase of unhealthy products and lower the amount of calories and saturated fat purchased (10), and, when compared to no labels, warning labels reduced the calorie and sugar content of purchased products, while other systems had no effect on purchasing (11). Bearing in mind the eye-catching packages that emphasize the quality of the alleged products, contrasting symbols for the inadequate nutrients are needed (9, 12).

Since 2017, Anvisa has started elaborating revisions and technical reports that sometimes consulted civil society. An increase in corporate political activities (CPA) of ultra-processed food and beverage industries stood out together with an agribusiness incurs on this subject, including the establishment of the coalition “Labeling Network” (13), an organization of influential Brazilian ultra-processed food and beverages industries. This group did not publicly disagree with the label update; however, it presented itself as the solution, initially defending the traffic-light labels. Evidence shows that this model lacks the efficiency to inform and discourage the purchase of harmful products (14–18).

During this long regulatory process in Brazil, many other countries conducted regulatory processes for nutrition food labels where a FoPNL was chosen, especially in Latin American countries. Since 2016 in Chile, packaged products have exhibited front-of-package black octagons, indicating excess of amounts of calories, sugar, sodium, and/or saturated fat (19). In addition, this warning label is currently approved and/or implemented Peru (20), Uruguay (21), Mexico (22), and Argentina (23).

Considering that scientific evidence and international experiences are not always enough to balance the influence of industries on political affairs, the involvement of civil organizations in the protection of a healthy diet and general health has been essential to guarantee the population's interests. In Brazil, the Alliance for Adequate and Healthy Diets launched the campaign “You have the right to know what you eat” in 2017. Due to Anvisa's slowness to go through the regulatory process, the Alliance launched, at the end of 2018, a second campaign with testimonies of doctors, fathers, mothers, young people, and people with NCD about the need for adequate food labeling, aiming to pressure Anvisa to open the public consultation (PC) about the nutrition labeling regulation. In 2019, during the PC, the Alliance encouraged the participation of the population with the third communication campaign “When you open your mouth, do not shut your eyes,” disclosing the civil society idea of the triangle warning labels on food packages.

In October 2020, Brazil approved the nutrition labeling of packaged foods, with an unprecedented FoPNL model in a magnifying glass format. Its design was modified and is smaller than the one presented in the PC with a considerable loss of graphic legibility and clarity. The design of the approved magnifying glass was never evaluated, with no scientific evidence that proves its efficiency. During the process of choosing, studies conducted under the ANVISA request tested the magnifying glass design presented in the PC and revealed that warning labels are more effective at being identified and facilitating the understanding of the nutrition composition, the perception of healthiness and shifting purchase intention (24, 25). Only one study showed a marginally better performance of the magnifying glass at improving purchase intentions when compared to triangles (26). Scientific evidence, published in 2021, after the approval of the Brazilian magnifying glass, showed that octagons were more effective in identifying the least harmful product, understanding the nutrient composition, and shifting purchase intentions (27).

The beginning of the implementation of the regulation is expected to October 2022 (28, 29). The approved model has a weaker nutrient profile than other countries (such as Chile) and the PAHO model, which means that unhealthy food products will not receive a magnifying glass, according to the Brazilian regulation. Added to this, the model defines that the product will receive just one magnifying glass independently of the number of critical nutrients (sugar, sodium, and/or saturated fat) present in the product. The nutrients will be listed beside or below the magnifying glass (Figure 1). The octagonal FoPNL, for example, determines one label for each nutrient.

**Figure 1:**
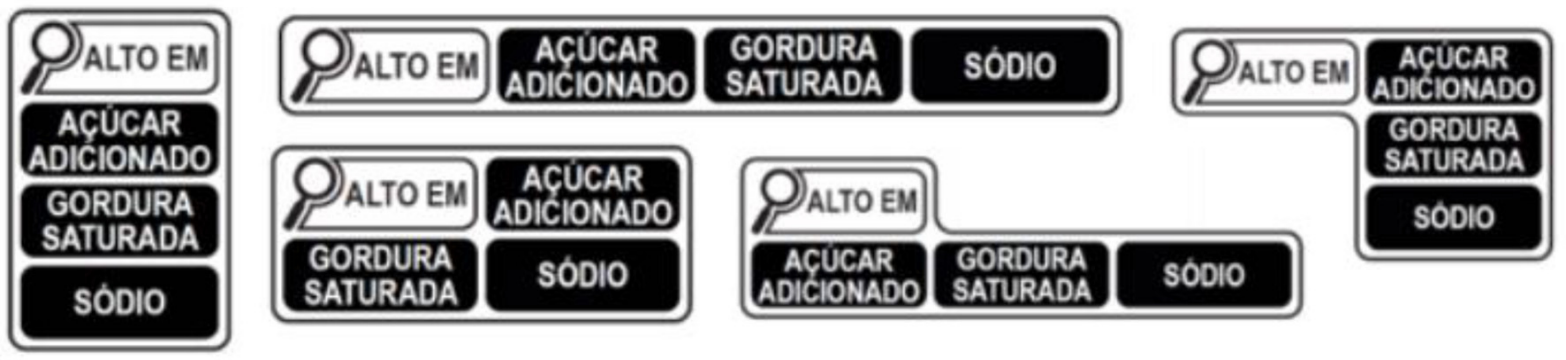
The FoPNL design model approved by Anvisa. Brazil, 2020. Source: Anvisa.

Based on the presented information, allied to the lack of scientific evidence of the model efficacy, the approved regulation was insufficient to meet both public health recommendations (3, 5) as the guarantee that all unhealthy food will receive the magnifying glass identification. Due to the challenging political scenario (a neoliberal government, concerned about financial market aspirations instead of the public health, with an ultra-right-wing position) and considerable interference of the industry during the regulatory process, the model approved was below the expected.

It took more than 6 years for Anvisa to decide on this regulatory process. During this time, the Brazilian political scenario went through several moments of instability that resulted in the replacement of Anvisa board directors, Health Ministers, and three presidents of the Republic. Besides the changes in power that slowed the process, the current government has a declared liberal position and defends commercial interests above public health.

In this context, besides the insufficiency of the regulation, the result of the regulatory process represents progress and recognition of the civil society work during this period, considering that they were able to defend the scientific evidence and the public health interest as a priority. The regulation approved by Anvisa represents one of the few losses of the food industry in the current government since they were not able to prevent the warning FoPNL. The food industry used the act in a way to reduce its scope, therefore, the effects of regulation acts (30).

After concluding this regulatory process, Brazil enhanced its health protection affairs, albeit industry interests are still a priority to detriment of public health interests. That impacts its capacity to fulfill its obligation of fully guaranteeing the right to health and food access. We advanced a step on the way to the right to information about the health risks from food products, but there are lots of changes and improvements to implement, and we will continue working toward the implementation of this regulation until every Brazilian has fully granted the right to know what he or she is eating.

## Author contributions

JG has designed and drafted the article. All of the others authors revised critically and approved the final version to be published.

## Funding

This work was funded by BRAZIL-RIIO-05B - Global Health Advocacy Incubator – Bloomberg Philanthropies.

## Conflict of interest

Author NR was employed by FIAN Brazil. Author PJ was employed by ACT Health Promotion. The remaining authors declare that the research was conducted in the absence of any commercial or financial relationships that could be construed as a potential conflict of interest.

## Publisher's note

All claims expressed in this article are solely those of the authors and do not necessarily represent those of their affiliated organizations, or those of the publisher, the editors and the reviewers. Any product that may be evaluated in this article, or claim that may be made by its manufacturer, is not guaranteed or endorsed by the publisher.
